# Modeling of the movement of rich gas in a porous medium in immiscible, near miscible and miscible conditions

**DOI:** 10.1038/s41598-023-33833-5

**Published:** 2023-04-21

**Authors:** Hossein Mehrjoo, Ali Safaei, Yousef Kazemzadeh, Masoud Riazi, Farid B. Cortés

**Affiliations:** 1 Department of Petroleum Engineering, Shahid Bahonar University of Kerman, Kerman, Iran; 2 Department of Petroleum Engineering, School of Chemical and Petroleum Engineering, Shiraz University, Shiraz, Iran; 3 Department of Petroleum Engineering, Faculty of Petroleum, Gas, and Petrochemical Engineering, Persian Gulf University, Bushehr, Iran; 4 Grupo de Investigación en Fenómenos de Superficie-Michael Polanyi, Departamento de Procesos y Energía, Facultad de Minas, Universidad Nacional de Colombia Sede Medellín, 050034 Medellín, Colombia

**Keywords:** Engineering, Chemical engineering

## Abstract

Gas injection is one of the most common enhanced oil recovery techniques in oil reservoirs. In this regard, pure gas, such as carbon dioxide (CO_2_), nitrogen (N_2_), and methane (CH_4_) was employed in EOR process. The performance of pure gases in EOR have been investigated numerically, but till now, numerical simulation of injection of rich gases has been scared. As rich gases are more economical and can result in acceptable oil recovery, numerical study of the performance of rich gases in EOR can be an interesting subject. Accordingly, in the present work the performance of rich gases in the gas injection process was investigated. Methane has been riched in liquefied petroleum gas (LPG), natural gas liquid (NGL), and Naphtha. Afterwards, the process of gas injection was simulated and the effect of injection fluids on the relative permeability, saturation profile of gas, and fractional flow of gas was studied. Our results showed that as naphtha is a heavier gas than the two other ones, IFT of oil-rich gas with naphtha is lower than other two systems. Based our results, gas oil ratio (GOR) and injection pressure did not affect the final performance of injection gas that has been riched in NGL and LPG. However, when GOR was 1.25 MSCF/STB, rich gas with naphtha moved with a higher speed in the domain and the relative permeability of each fluid and fractional flow of gas were affected. The same result was achieved at higher injection pressure. When injection pressure was 2000 psi, movement of gas with higher speed in the domain, alteration of relative permeability and changes in the fractional flow of gas were obvious. Therefore, based on our result, injection of naphtha with low pressure and high GOR was suggested for considered oil.

## Introduction

As hydrocarbon demand stays high and its production from the field is declining, in the future, the hydrocarbon demand will outrun the world's hydrocarbon production^1^. Therefore, the significance of using new techniques to produce more hydrocarbon is very important^1^. Different enhanced oil recovery (EOR) techniques can be used for the production of more hydrocarbon, one of the commonly used EOR methods is gas injection, especially for light oil reservoir^1–3^. Poor injectivity and sensitivity issues are known as limitations in water flooding, which can be overcome in the gas injection process^4^. As most of injection gases are greenhouse gas, this technique is known as an environmentally friendly method^4^. Both natural and non-natural gas can be injected to enhance the production of oil in mature fields. Carbon dioxide (CO_2_), associated gas, air, and nitrogen (N_2_) are the most used gases in gas flooding^4,5^. Because of its physical properties, viscosity reduction and oil swelling can be made well with CO_2_. In addition, the existence of CO_2_ in some formations can result in the dissolution of limestone minerals and subsequently increase formation injectivity^6–9^. Accessibility and low cost of air caused this gas to become one of the interesting injection fluids in gas flooding^10^. The main issue in air injection is its oxygen content, as it can create a threat to the safety of the operation process^11^. The main feature of associated gas is its high solubility in reservoir fluid. Hence, it can generate a miscible flooding process, but due to its high cost, it will be injected with other natural gas^12^. The performance of flue gas in the reservoirs that contain heavy crude oil and have thin-pay is acceptable^13^. Because of the physical properties of flue gas, it is unlikely to form miscible phase with reservoir fluid; therefore, massive amount of injected gas remains as a free gas^13^. Gas can be injected in miscible, immiscible, and near miscible conditions, which depends on the reservoir temperature and pressure, hydrocarbon components of reservoir, and type of injection gas. High reservoir pressure and gas with low minimum miscibility pressure (MMP) resulted in miscible conditions, and injected fluid and reservoir fluid created a single phase. In this process, the viscosity of the oil will be reduced, and oil swelling will be occurred^14^. In an immiscible process, the oil displacement is controlled by capillary pressure and relative permeability^14^. Unlike the miscible process, in immiscible injection, only part of the gas will be dissolved in the reservoir oil^14^.

As the gas injection is known as an interesting EOR technique, researchers have studied this method experimentally as well as numerically. Karimaie et al.^15^ investigated the process of gas injection in a fractured reservoir. They showed that CO_2_ resulted in more oil production than N_2_ at high pressure and temperature. Chukwudeme and Hamouda^16^ conducted a series of experiments to study miscible CO_2_ flooding. Based on their experiments, compared with asphaltenic oil, CO_2_ flooding resulted in more oil recovery for non-asphaltenic oil. The performance of CO_2_ in tight reservoirs was studied by Arshad et al.^17^. Oil recoveries of 87–97% were achieved in their experiments. In 2014, Bhoendie et al.^18^ conducted a series of core flooding tests to investigate the potential of some N_2_ and CO_2_ injections. They showed that both CO_2_ and N_2_ WAG caused the highest oil recovery. Injection of N_2_ after water flooding had a higher recovery than CO_2_ flooding after water injection. In the other hand, in gas injection phase CO_2_ had higher oil production compared with N_2_. In order to investigate immiscible N_2_ flooding, Janssen et al.^19^ conducted five series of experiments: continuous N_2_ injection at increasing backpressures, continuous N_2_ injection at 5 and 10 bar backpressure, continuous N_2_ injection after water flooding, and water alternative gas^20^ injection. Their experiments illustrated that WAG injection resulted in more oil recovery, among other schemes. Huang et al.^4^ investigated the performance of CO_2_, deoxygenated air, associated gas, and fuel gas in the tertiary recovery process. Based on their experiments, the performance of continuous gas injection was better than WAG injection. In addition, among the above-mentioned gas and in continuous gas injection, associated gas has better performance in oil recovery. They showed that in the WAG process, the performance of CO_2_ is much better than the three other gases. The experimental study was conducted by Khather et al.^7^ to study the impact of CO_2_ injection on both oil recovery and petrophysics. They reported that the injection of CO_2_ after water flooding resulted in higher oil recovery. Based on their experiment, a reduction in permeability of samples occurred after the CO_2_ injection. Different mechanisms such as mineral precipitation/dissolution, resin precipitation, compaction, and asphaltene precipitation can be the reasons for this reduction^7^. The performance of associate gas huff-n-puff in organic-rich shale core was evaluated by Shilov et al.^21^. Based on their observation in non-miscible conditions, oil recovery increased from 29 to 79.55%. While in the near miscible condition, the oil recovery was improved from 41 to 88.4%.

Besides extensive experimental study in the gas injection process, there is various numerical study. Hoteit and Firoozabadi^22^ developed a numerical model for the gas injection process in the fractured reservoir that considers the physical diffusion of the multicomponent mixture. Based on their results, away from miscibility pressure, the effect of diffusion on the recovery was high. They also investigated their model for fractured gas condensate and showed that diffusion affected the condensate recovery. Alfarge et al.^23^ investigated the numerical simulation study on miscible EOR techniques for enhancing oil recovery in shale oil reservoirs. Their simulation results showed the significance of molecular diffusion in a gas injection on EOR for the studied reservoirs. Zhong et al.^24^ studied a numerical simulation of the WAG process for optimizing carbon storage and EOR. Their results showed that injection of WAG caused the mobility ratio of water and oil to decrease and swept coefficient to increase, the total recovery of the reservoir after WAG injection increased. In terms of CO_2_ storage, a low WAG time ratio was better. However, in terms of oil recovery, a high WAG time ratio was acceptable. They also showed that higher oil recovery could be achieved because of the higher injection rate of CO_2_. Shilov et al.^21^ investigated the performance of associated gas huff-n puff and validated their observation with numerical study. Based on their numerical study, injection pressure influenced the oil recovery, especially in near miscible and miscible conditions.

As there is a high demand for oil, unconventional reservoir, such as shale oil, tight oil, coal seam gas/coal bed-methane, shale gas, basin-center gas, gas hydrates, tight gas, chalk, and tar sand, have become main source of oil production. There is some substantial differences between conventional and unconventional reservoirs. Large volume of formations that charged with hydrocarbon are exist in unconventional reservoirs. Buoyancy and gravity of reservoir fluids do not affect the production of these type of reservoirs. Reservoir and source rock in unconventional reservoirs are coexist, while in conventional reservoir they are separated form each other. In this regard, special methods was employed to produce for these type of reservoirs^25^. According to important role of unconventional reservoir in energy production, several researchers investigated the performance of different EOR method in theses reservoirs. As well as conventional reservoirs, gas injection is one of interesting EOR method in unconventional reservoirs. Therefore, many experimental and numerical investigations have been conducted to study gas injection technique in unconventional reservoirs^26–34^. The potential of cyclic CO_2_ injection for oil recovery in shale reservoirs was investigated experimentally by Gamadi et al.^32^. They showed that miscible CO_2_ affected the oil recovery more than immiscible CO_2_ injection. In addition, based on their experiment, the injection of CO_2_ at MMP did not have a strong effect on the final oil recovery. Meng et al.^26^ proposed a huff-*n*-puff gas injection for improving recovery from shale gas-condensate reservoirs. Based on their experiments, they introduced huff-*n*-puff gas injection as a suitable technique for enhancing gas condensate recovery in shale gas reservoirs. An experimental study to investigate the potential of the huff-*n*-puff gas injection method for recovery in shale gas-condensate reservoirs was conducted by Meng et al.^27^. Then to verify their experimental results, the numerical model was developed. Based on their results, the huff-*n*-puff technique is a suitable method for recovery in shale gas-condensate reservoirs. In order to study the mechanism of CO_2_ EOR in the unconventional reservoirs, a numerical study was conducted by Zhang et al.^28^. Their study indicated that higher oil production is achieved as a result of CO_2_ injection in these types of reservoirs via hydraulic fractures. Based on their results, among different gas mechanisms, diffusion played a minimal role, while multi-contact miscibility was a dominant mechanism.

As shown in the literature review, the process of gas injection is one of the interesting methods. Therefore, in the present study the effect and performance of these gases was investigated. New developed code can investigate the effect of each rich gases on IFT of system, the fractional flow, gas saturation profile and relative permeability of gas and oil and based on these output can be achieved comprehensive information about the performance of these gases. In addition, based on considered crude oil and developed code in the present study, sensitivity analysis can be conducted on GOR, and injection pressure, and suitable injection rich gas, GOR, and injection pressure for introduced crude oil can be suggested. It is worth noting to mention that developed code considered the effect of porous media. In order to develop aforementioned code, first methane has been riched in Naphtha, LPG, and NGL and the composition of rich gases was determined, then developed codes was validated with available experimental and numerical data. Our developed code includes the estimation of compressibility factor, gas-oil ratio, and formation volume factor. Then based on empirical correlation, the IFT of the system was predicted. After modification of the relative permeability and viscosity of each fluid, the modified Buckley-Leverett equation was used to determine the saturation profile and fractional flow of gas. After that, the results of the simulation are presented and discussed. In the end, the conclusions of this work are presented.

## Method of work

### Mathematical model

**Figure 1:**
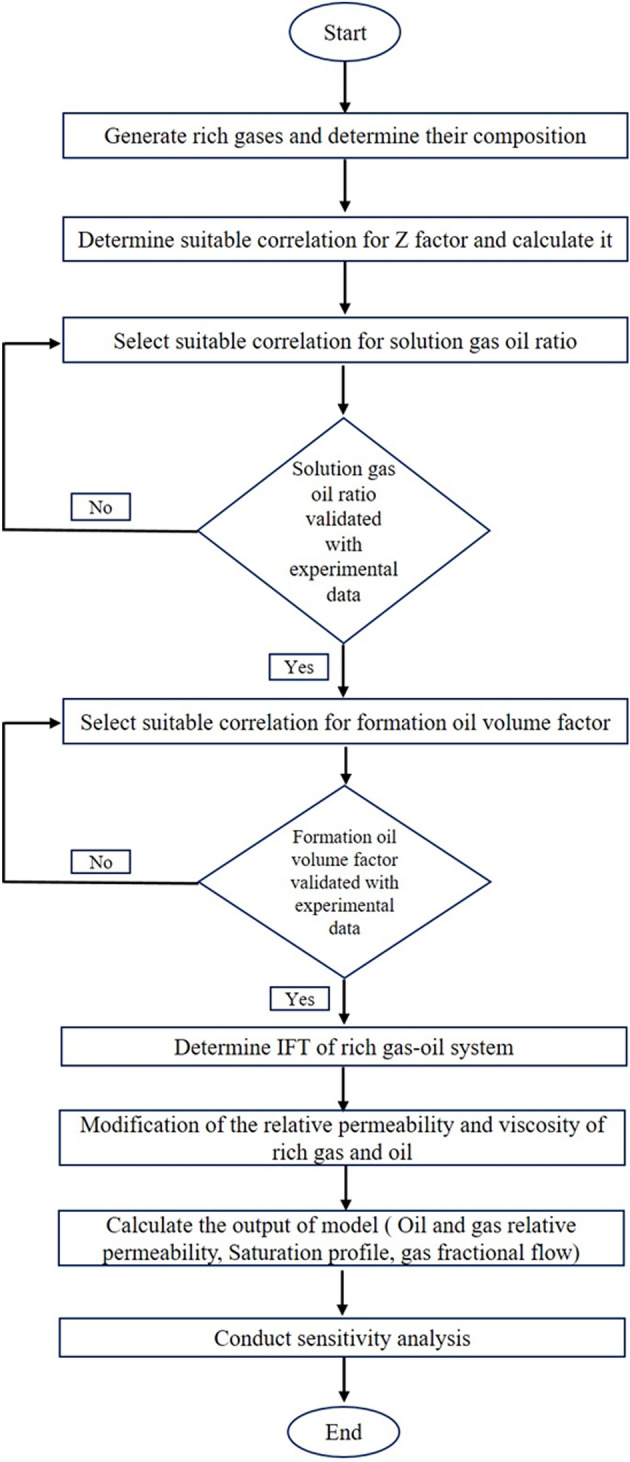
The Flow chart of the present simulation.

This study contains four steps: (1) mixing of methane with LPG, NGL, and Naphtha and generation of rich gas, (2) calculation of interfacial tension (IFT) of rich gas-oil system, (3) modification of relative permeability and viscosity of rich gas and oil, (4) determination of the model output, i.e., relative permeability, rich gas saturation and fractional flow of rich gas (Fig. 1).

At the first step, NGL, LPG, and Naphtha were mixed with methane at three different GORs of 1.25, 2.5, and 5 MSCF/STB. Tables 1, 2 and 3 presented the final composition of rich gases.

**Table 1:** The composition of rich gas with LPG.

Composition	Mole fraction (percent)
GOR = 1.25 MSCF/STB	
C_1_	94.565
C_2_	4.9771
C_3_	0.21998
C_4_	0.22914
C_5_	0.0091656
GOR = 2.5 MSCF/STB	
C_1_	94.782
C_2_	4.9885
C_3_	0.11024
C_4_	0.11483
C_5_	0.0045933
GOR = 5 MSCF/STB	
C_1_	94.891
C_2_	4.9943
C_3_	0.055183
C_4_	0.057483
C_5_	0.0022993

**Table 2:** The composition of rich gas with NGL.

Composition	Mole fraction (percent)
GOR = 1.25 MSCF/STB	
H_2_S	1.5081 × 10^–5^
CO_2_	0.0012668
C_1_	94.565
C_2_	5.095
C_3_	0.16113
i-C_4_	0.034257
n-C_4_	0.074075
i-C_5_	0.020907
n-C_5_	0.022023
C_6_	0.014765
C_7_	0.0051138
C_8_	0.0032772
C_9_	0.0022089
C_10_	0.0011579
GOR = 2.5 MSCF/STB	
H_2_S	7.5586 × 10^–6^
CO_2_	0.00063493
C_1_	94.782
C_2_	5.0476
C_3_	0.080759
i-C_4_	0.01717
n-C_4_	0.037127
i-C_5_	0.010479
n-C_5_	0.011038
C_6_	0.0074001
C_7_	0.0025631
C_8_	0.0016426
C_9_	0.0011071
C_10_	0.00058036
GOR = 5 MSCF/STB	
H_2_S	7.6831 × 10^–6^
CO_2_	0.00083352
C_1_	90.713
C_2_	9.0018
C_3_	0.10623
i-C_4_	0.029769
n-C_4_	0.064371
i-C_5_	0.022552
n-C_5_	0.023757
C_6_	0.018542
C_7_	0.0073397
C_8_	0.0052427
C_9_	0.0039959
C_10_	0.0023198

**Table 3:** The composition of rich gas with Naphtha.

Mole fraction (percent)	Mole fraction (percent)
GOR = 1.25 MSCF/STB	
C_1_	53.034
C_2_	0
C_3_	0
i-C_4_	1.0098
n-C_4_	0.051663
i-C_5_	19.773
n-C_5_	6.2465
C_6_	18.664
C_7_	1.2211
GOR = 2.5 MSCF/STB	
C_1_	69.31
C_2_	0
C_3_	0
i-C_4_	0.65984
n-C_4_	0.033759
i-C_5_	12.921
n-C_5_	4.0818
C_6_	12.196
C_7_	0.79794
GOR = 5 MSCF/STB	
C_1_	90.713
C_2_	9.0018
C_3_	0.10623
i-C_4_	0.029769
n-C_4_	0.064371
i-C_5_	0.022552
n-C_5_	0.023757
C_6_	0.018542
C_7_	0.0073397

In the second step, the IFT of rich gas-oil must be determined. There are several methods for calculation of the IFT of the system. Ramey's correlation^35^ was used for the IFT of the system:

1 $${\sigma }_{go}^{1/4}={P}_{o}\left({x}_{o}\frac{{\rho }_{o}}{{M}_{og}}-{y}_{o}\frac{{\rho }_{g}}{{M}_{go}}\right)-{P}_{g}\left({x}_{g}\frac{{\rho }_{o}}{{M}_{og}}-{y}_{g}\frac{{\rho }_{g}}{{M}_{go}}\right).$$

In above equation, ${\sigma }_{go}$ is the IFT of gas-oil system. ${x}_{g}$, ${x}_{o}$, ${y}_{g}$, and ${y}_{o}$ present mole fraction of components in the oil and gas phase, respectively. The density of gas and oil is shown by ${\rho }_{g}$ and ${\rho }_{o}$, respectively. ${M}_{go}$ and ${M}_{og}$ show the average molecular weight of the gas and oil phase, respectively. In addition, Parachor equation for gas and oil phase is presented by ${P}_{g}$ and ${P}_{o}$, respectively. Equations (2)–(13) was used to calculated considered parameters for Ramey's correlation:

2 $${M}_{g}=28.97\times {\gamma }_{g},$$

3 $${M}_{o}=6084/({\gamma }_{API}-5.9),$$

4 $${P}_{g}=25.2+2.86{M}_{g},$$

5 $${P}_{o}=\left(2.376+0.0102{\gamma }_{API}\right){M}_{o},$$

6 $${x}_{o}={[1+\frac{7.521\times {10}^{-6}{R}_{s}{M}_{o}}{{\gamma }_{o}}]}^{-1},$$

7 $${x}_{g}=1-{x}_{o},$$

8 $${y}_{o}={[1+\frac{7.521\times {10}^{-6}{M}_{o}}{{\gamma }_{o}{r}_{v}}]}^{-1},$$

9 $${y}_{g}=1-{y}_{o},$$

10 $${\rho }_{g}=9.3184\times {10}^{-2}\frac{P{M}_{go}}{62.4*ZT},$$

11 $${\rho }_{o}=\frac{{\gamma }_{o}+2.179\times {10}^{-4}{\gamma }_{g}{R}_{s}}{{B}_{o}},$$

12 $${M}_{go}={y}_{o}{M}_{o}+{y}_{g}{M}_{g},$$

13 $${M}_{og}={x}_{o}{M}_{o}+{x}_{g}{M}_{g},$$

where ${M}_{o}$ and ${M}_{g}$ show molecular weight of oil and gas phase. Compressibility factor and solution gas-oil ratio are denoted by $Z$, and ${R}_{s}$. Vaporized oil in the gas phase which considered 0 in the present study is shown by ${r}_{v}$.

In order to determine the IFT of the system, critical pressure and temperature, solution gas-oil ratio, formation volume factor, and compressibility factor must be determined. To this end, Sutton's correlation^36^ for critical temperature and pressure, Brill and Beggs' correlation^37^ for compressibility factor, and Standing's correlation^38^ for solution gas-oil ratio and oil formation volume factor were used.

14 $${T}_{pc}=169.2+349.5\times {\gamma }_{g}-74\times {\gamma }_{g}^{2},$$

15 $${T}_{pr}=\frac{T}{{T}_{pc}},$$

16 $$tpr=\frac{1}{{T}_{pr}},$$

17 $${P}_{pc}=756.8-131.07\times {\gamma }_{g}-3.6\times {\gamma }_{g}^{2},$$

18 $${P}_{pr}=\frac{P}{{P}_{pc}},$$

19 $$A=1.39{({T}_{pr}-0.92)}^{0.5}-0.36{T}_{pr}-0.10,$$

20 $$B=\left(0.62-0.23{T}_{pr}\right){P}_{pr}+\left(\frac{0.066}{{T}_{pr}-0.86}-0.037\right){P}_{pr}^{2}+\frac{0.32{P}_{pr}^{2}}{{10}^{E}},$$

21 $$C=0.132-0.32\mathrm{log}\left({T}_{pr}\right),$$

22 $$D={10}^{F},$$

23 $$E=9\left({T}_{pr}-1\right),$$

24 $$F=0.3106-0.49{T}_{pr}+0.1824{T}_{pr}^{2},$$

25 $$Z=A+\frac{1-A}{{e}^{B}}+C{p}_{pr}^{D},$$

26 $$a=0.00091\left(T-460\right)-0.0125{\gamma }_{API},$$

27 $${R}_{s}={\gamma }_{g}{[(\frac{p}{18.2}+1.4){10}^{a}]}^{1.2048},$$

28 $${B}_{o}=0.9759+0.000120{[{R}_{s}{\left(\frac{{\gamma }_{g}}{{\gamma }_{o}}\right)}^{0.5}+1.25(T-460)]}^{1.2}.$$

Pseudocritical temperature and pressure are shown by ${T}_{pc}$ and ${P}_{pc}$. In addition, pseudoreduced pressure and temperature are illustrated by ${P}_{pr}$ and ${T}_{pr}$. ${\gamma }_{g}$ and ${\gamma }_{o}$ show the specific gravity of gas and oil, respectively. ${\gamma }_{o}$ and ${\gamma }_{API}$ can be related to each other through ${\gamma }_{API}=\frac{141.5}{{\gamma }_{o}}-131.5$. Oil formation volume factor is denoted by ${B}_{o}$, respectively. In the above equation, temperature and pressure are shown by $T$ and $P$, respectively. In addition, A-F are the constants for Brill and Beggs' correlation.

One of the main parts of this numerical study is the modification of relative permeability. There are several methods for modification of relative permeability. However, the method introduced by Coats^39^ is known as a suitable method and used in the present study. In this method, the effect of IFT was involved in the modification of relative permeability. The effect of IFT was considered through the relative permeability interpolation parameter, ${F}_{k}$:

29 $${F}_{K}=min\left[1,{\left(\frac{\sigma }{{\sigma }_{0}}\right)}^{N}\right],$$

30 $$N=\frac{1}{{n}_{l}}.$$

Now by using the Corey–Brook correlation^40^, the relative permeability of each phase was determined as follows:

31 $${S}_{gi}={F}_{K}\times {S}_{gi}^{imm},$$

32 $${S}_{or}={F}_{K}\times {S}_{or}^{imm},$$

33 $${S}_{gn}=\frac{{S}_{g}-{S}_{gi}}{1-{S}_{gi}-{S}_{or}},$$

34 $${K}_{rg}^{imm}={K}_{rg}\times {S}_{gn}^{ng},$$

35 $${K}_{ro}^{imm}={K}_{ro}\times {\left(1-{S}_{gn}\right)}^{{n}_{o}},$$

36 $${K}_{rg}^{mis}={S}_{gn}^{{n}_{m}},$$

37 $${K}_{ro}^{mis}={(1-{S}_{gn})}^{{n}_{m}}.$$

Then by using the ${F}_{k}$, the miscible and immiscible relative permeability of each phase was measured.

38 $${K}_{RG}={\left(1-{F}_{K}\right)\times {K}_{rg}^{mis}+F}_{K}\times {K}_{rg}^{imm},$$

39 $${K}_{RO}={\left(1-{F}_{K}\right)\times {K}_{ro}^{mis}+ F}_{K}\times {K}_{ro}^{imm},$$

where ${K}_{RO}$, and ${K}_{RG}$ show modified oil and gas relative permeability, respectively. In above equations, the immiscible and miscible oil relative permeability is presented by ${K}_{ro}^{imm}$ and ${K}_{ro}^{mis}$, respectively. In addition, ${K}_{rg}^{imm}$, and ${K}_{rg}^{mis}$ are immiscible and miscible gas relative permeability, respectively. ${\sigma }_{0}$, $\sigma$, and ${n}_{l}$ illustrate the IFT at MMP, IFT at intended pressure, and read in exponent, respectively. ${S}_{or}$, ${S}_{or}^{imm}$, ${S}_{g}$, ${S}_{gi}$, and ${S}_{gi}^{imm}$ show modified residual oil saturation, residual oil saturation at immiscible conditions, gas saturation, modified irreducible gas saturation, and irreducible gas saturation at immiscible conditions. ${K}_{ro}$ and ${K}_{rg}$ illustrated the relative permeability of oil and gas at irreducible gas and residual oil saturation, respectively. In addition, gas and oil exponent for Brooks–Corey functions is shown by ${n}_{g}$ and ${n}_{o}$, respectively. ${n}_{m}$ is relative permeability index and in the present study is considered 1.1.

Modification of viscosity is another step of this study. For this purpose, Todd–Longstaff^41^ model was used:

40 $${\mu }_{geff}={\mu }_{g}^{1-\omega }\times {\mu }_{m}^{\omega },$$

41 $${\mu }_{oeff}={\mu }_{o}^{1-\omega }\times {\mu }_{m}^{\omega },$$

42 $${(\frac{1}{{\mu }_{m}})}^\frac{1}{4}=\frac{{S}_{g}^{^{\prime}}}{{S}_{n}^{^{\prime}}}{(\frac{1}{{\mu }_{g}})}^\frac{1}{4}+\frac{{S}_{o}^{^{\prime}}}{{S}_{n}^{^{\prime}}}{(\frac{1}{{\mu }_{o}})}^\frac{1}{4},$$

43 $${S}_{g}^{^{\prime}}={S}_{g}-{S}_{gi},$$

44 $${S}_{o}^{^{\prime}}={S}_{o}-{S}_{or},$$

45 $${S}_{n}^{^{\prime}}={S}_{o}^{^{\prime}}-{S}_{g}^{^{\prime}}.$$

In this study, the mixing factor of viscosity ($\omega$) is 1/3. ${\mu }_{geff}$, ${\mu }_{oeff}$, and ${\mu }_{m}$ show gas and oil effective viscosity, and mixing viscosity, respectively.

In order to study the process of gas injection and determine the output of the model, the Buckley–Leverett model was used ^42^:

46 $${f}_{g}=\frac{{S}_{g}^{2}}{{S}_{g}^{2}+{(1-{S}_{g})}^{2}VR}.$$

In Eq. (46), $V$ shows the viscosity ratio and fractional flow for the gas phase are presented by ${f}_{g}$. Derivative of the fractional flow of gas phase to gas saturation is determined through the following equation:

47 $$\frac{{df}_{g}}{{dS}_{g}}=\frac{2V({S}_{g}-1){S}_{g}}{{(V{\left({S}_{g}-1\right)}^{2}+{S}_{g}^{2})}^{2}}.$$

Dimensionless pore volume and distance transferred by a specific ${S}_{g}$ contour are measured through the following equations:

48 $$PVI=\frac{{q}_{t}t}{L\phi Area},$$

49 $${x}_{{S}_{g}}=PVI\times L{\left(\frac{{df}_{g}}{{dS}_{g}}\right)}_{{S}_{g}}.$$

In the above equation, dimensionless pore volume, porosity, length of the domain, cross-section area, total injection rate, injection time, and moving distance by a specific ${S}_{g}$ contour are shown by $PVI$, $\phi$, $L$, $Area$, ${q}_{t}$, $t$, and ${x}_{{S}_{g}}$, respectively.

## Results and discussion

### Numerical simulation

Simulation parameter that used in the present study is shown in Table 4:

**Table 4:** Parameters for simulation of IFT and gas injection.

Inputs parameters	Value
Oil viscosity (${\mu }_{o}$)	1.81 mPa s
API of reservoir oil ($API$)	19.96
Oil relative permeability at irreducible gas saturation ($KRO$)	0.9
Residual oil saturation (${S}_{or}$)	0.24
Initial saturation of oil (${S}_{oi}$)	0.95
${n}_{o}$	2.1079
Bubble point pressure (${P}_{b}$)	1379 psi
Gas viscosity (${\mu }_{g}$)	0.035 mPa s
Gas relative permeability at residual oil saturation ($KRG$)	0.6181
Initial saturation of gas (${S}_{gi}$)	0.05
${n}_{g}$	2.9852
Reservoir temperature (${T}_{res}$)	643.77 R
Length of the simulated domain ($L$)	200 m
Dimensionless pore volume	0.015
N	1/4

**Figure 2:**
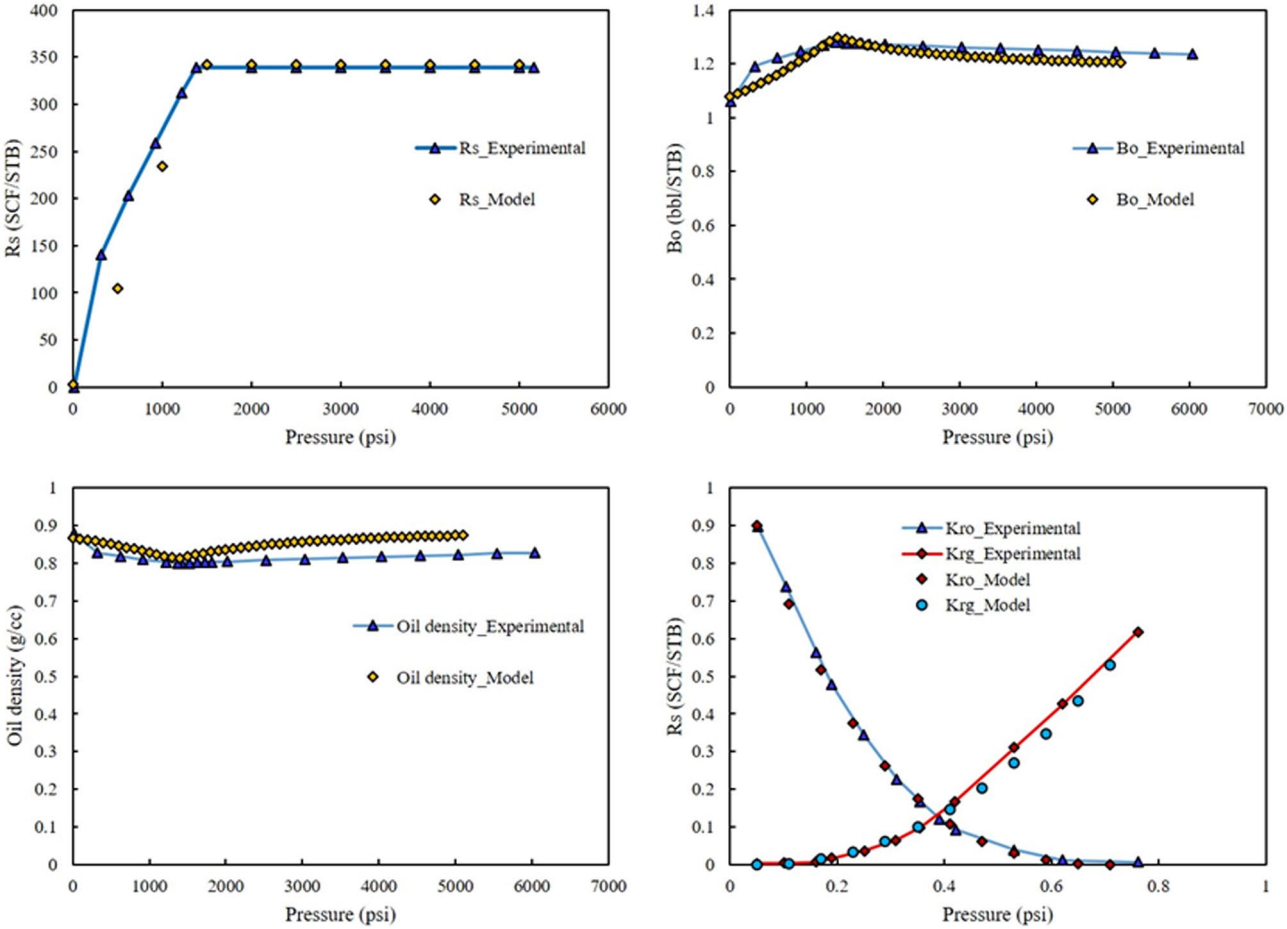
Comparison of the simulated value solution gas-oil ratio, oil formation volume factor, oil density, and relative gas and oil permeability.

**Figure 3:**
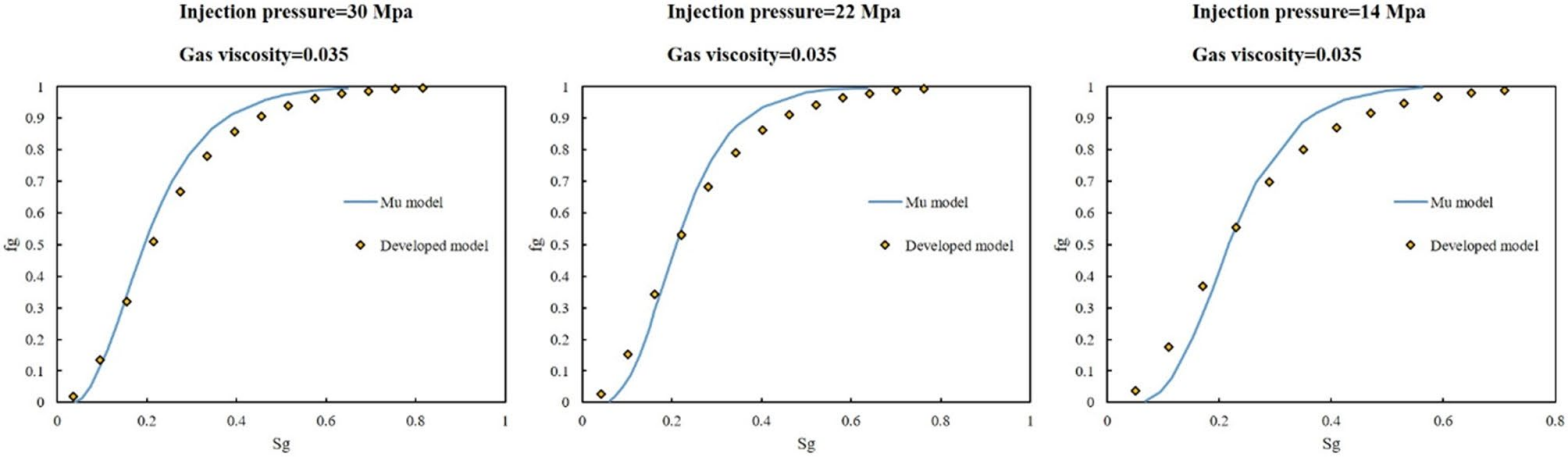
Comparison of simulated value of fractional flow at gas viscosity of 0.035 mPa s three injection pressures of 30, 22, and 14 MPa with the results of Mu et al.^43^.

Some numerical and experimental study was used to investigate the performance of correlations used in the present study. As shown in Figs. 2 and 3, the correlations had acceptable performance.

### Effect of injected rich gas on the output of the model

**Figure 4:**
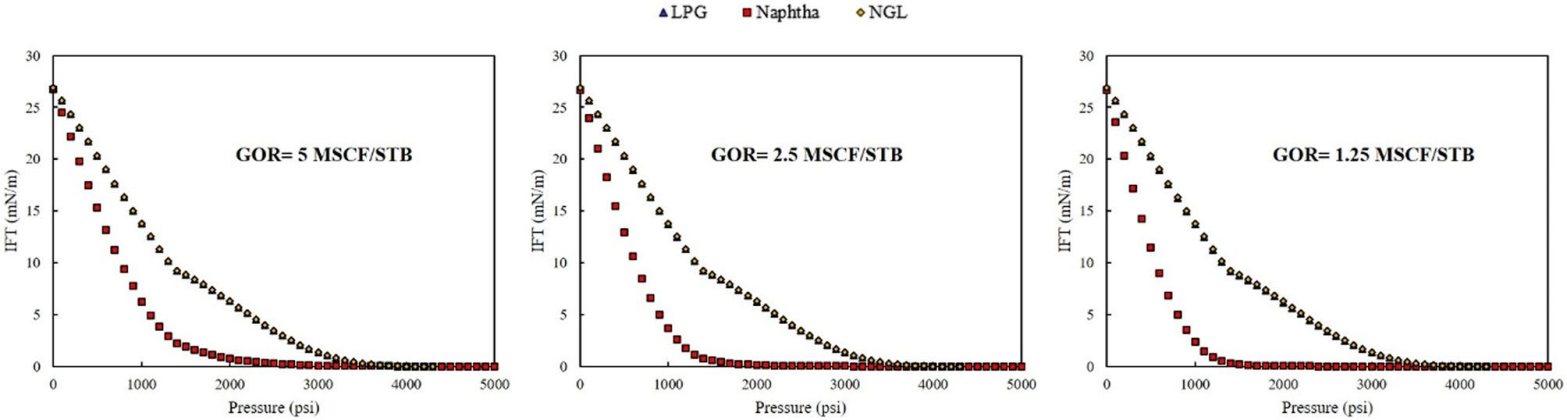
IFT of rich gas-oil system for three GORs of 1.25, 2.5, and 5 Mscf/STB.

Three rich gas was used as an injection fluid in the present study. The effect on the relative permeability, saturation profile, and fractional flow was investigated in this section. Figure 4 shows the IFT of the rich gas-oil system at different GOR.

**Figure 5:**
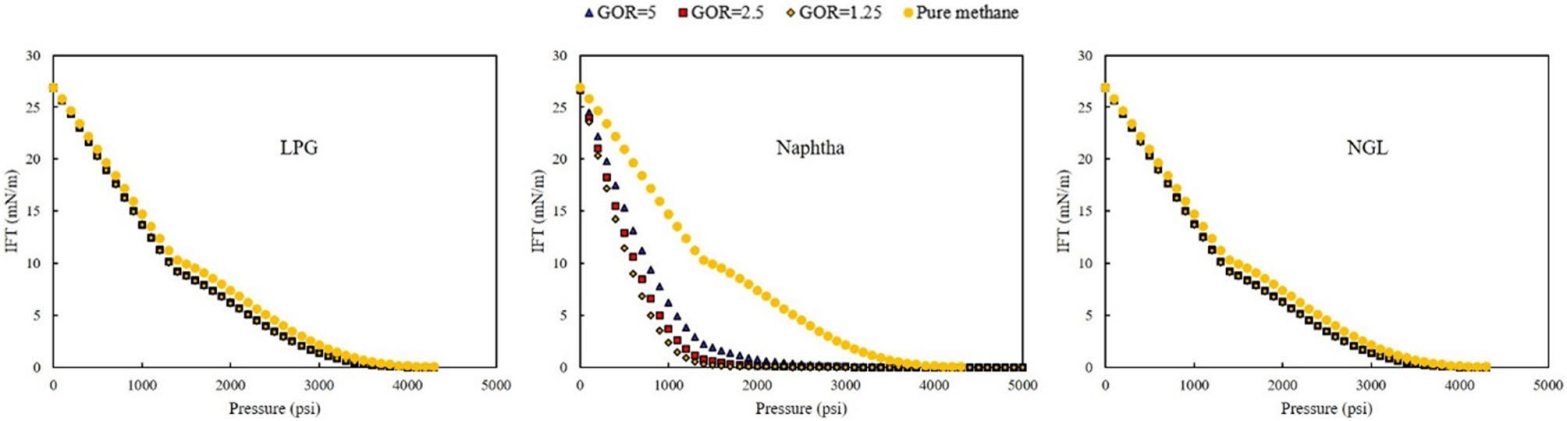
Comparison of IFT of methane-oil system and each rich gas-oil system at different GOR.

As shown in Fig. 4, when injected gas was rich gas with NGL and LPG, the IFT of the system was more than the condition that injected gas was a rich gas with Naphtha. The main reason for this phenomenon was that Naphtha is a heavy gas; therefore, lower IFT for the oil-rich gas systems was achieved. The percentage of methane in the composition of LPG and NGL was higher than other components; hence, as shown in Fig. 5, the IFT of rich gas-oil system for rich gas with NGL and LPG was closed to the IFT of methane-oil system.

### Effect of gas-oil ratio

**Figure 6:**
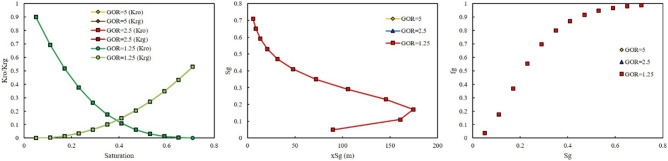
Effect of GOR of LPG on relative permeability, saturation profile, and fractional flow curve at an injection pressure of 500 psi.

**Figure 7:**
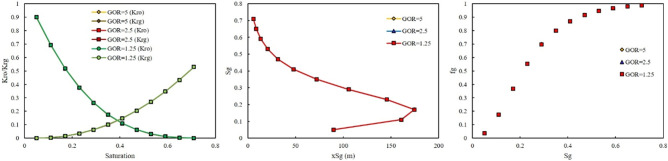
Effect of GOR of LPG on relative permeability, saturation profile, and fractional flow curve at an injection pressure of 3115 psi.

As previously mentioned, rich gas were made with different GORs. The effect of GOR on the relative permeability, saturation profile, and fractional flow for each rich gas was investigated in this section. Figures 6 and 7 show the effect of GOR at two injection pressures of 500 and 3115 psi on the output of the model once gas-rich with LPG was used as an injection gas. When LPG was used for rich gas, the final composition of gas was not severely changed. Therefore, the GOR cannot affect the output of the model, as shown in Figs. 6 and 7.

**Figure 8:**
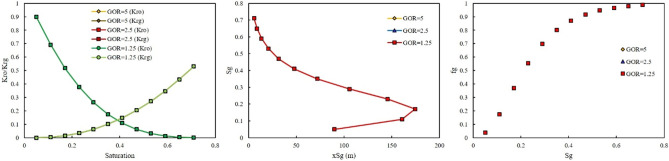
Effect of GOR of Naphtha on relative permeability, saturation profile, and fractional flow curve at an injection pressure of 500 psi.

**Figure 9:**
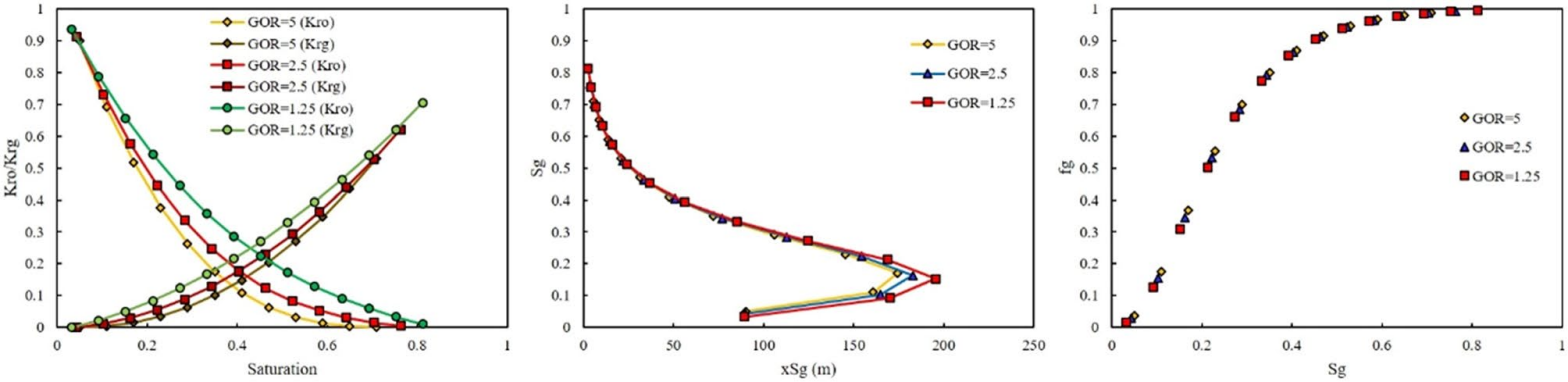
Effect of GOR of Naphtha on relative permeability, saturation profile, and fractional flow curve at an injection pressure of 1500 psi.

**Figure 10:**
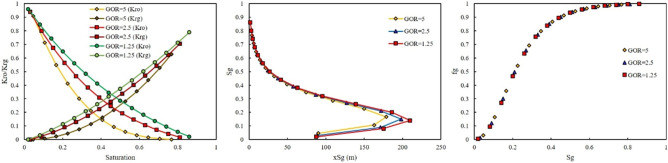
Effect of GOR of Naphtha on relative permeability, saturation profile, and fractional flow curve at an injection pressure of 2000 psi.

In the second scenario, a gas was riched in Naphtha was injected. In this scenario, three-injection pressure of 500, 1500, and 2000 psi was used. As shown in Fig. 8, at low injection pressure, GOR cannot affect the relative permeability curve, saturation profile curve, and fractional flow curve. However, by increasing the injection pressure, the effect of GOR appeared. At injection pressure of 1500 psi, higher GOR, 5 MSCF/STB, resulted in shifting the relative permeability of oil to the left side, increase, and relative permeability of gas to the right side, decrease (Fig. 9). In addition, lower GOR, 1.25 MSCF/STB, caused gas to move faster in the domain (Fig. 9), and the effect of GOR on the fractional flow curve was evident in Fig. 9. Higher GOR resulted in higher fractional flow of gas and consequently lower oil production will be expected. The main reason for this phenomenon was that at lower GOR, the amount of methane in the gas composition was higher than in other GORs. Therefore, the gas moved faster in the domain, shifting the relative permeability of oil to the right side and the relative permeability of gas to the left side. In other words, at high GOR the gas became heavy and its relative permeability will be decrease, therefore it moved with lower velocity in the domain and breakthrough will not be occurred and more oil will be produced. The same results were observed at higher injection pressure, 2000 psi (Fig. 10).

**Figure 11:**
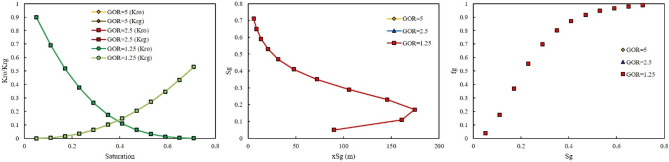
Effect of GOR of NGL on relative permeability, saturation profile, and fractional flow curve at an injection pressure of 500 psi.

**Figure 12:**
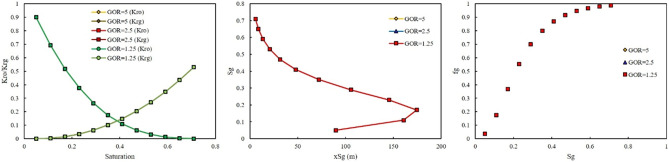
Effect of GOR of NGL on relative permeability, saturation profile, and fractional flow curve at an injection pressure of 3000 psi.

The third injection gas was rich with NGL. The effect of GOR at two injection pressure of 500 and 3000 psi was investigated. The same as LPG, the effect of GOR on the output of the model at two injection pressures were not observed (Figs. 11, 12).

### Effect of injection pressure

**Figure 13:**
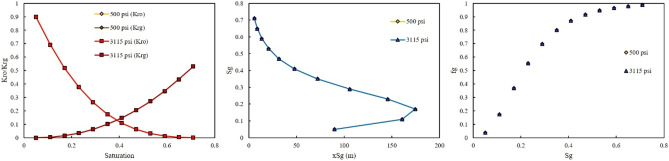
Effect of injection pressure of LPG on relative permeability, saturation profile, and fractional flow curve at GOR 2.5 MSCF/STB.

**Figure 14:**
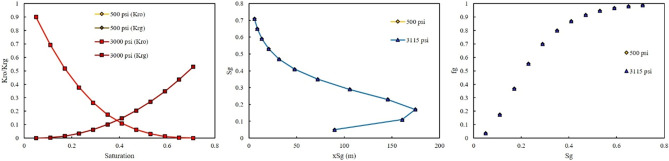
Effect of injection pressure of NGL on the (**a**) relative permeability, (**b**) saturation profile, and (**c**) fractional flow curve at GOR 2.5 MSCF/STB.

**Figure 15:**
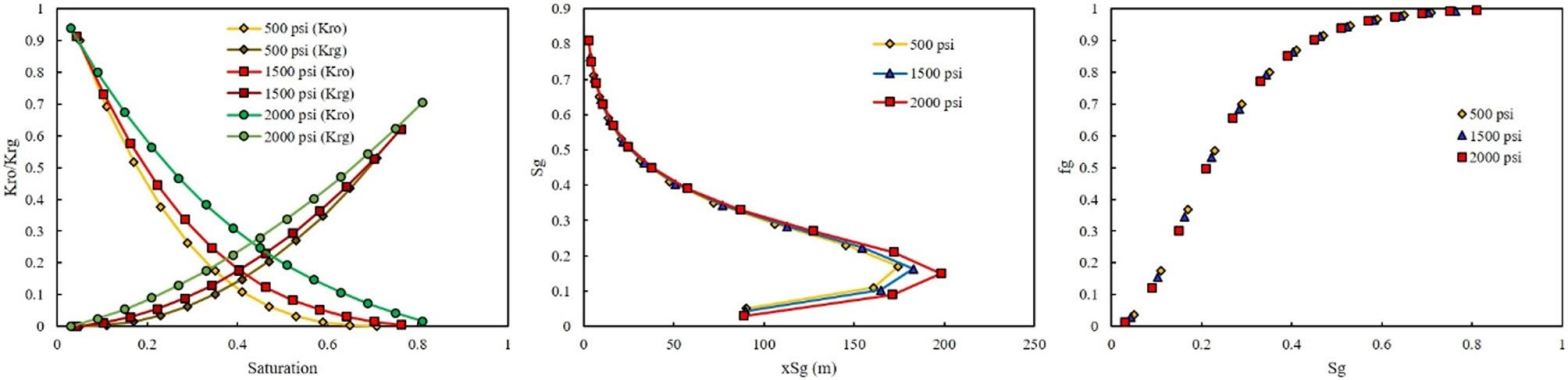
Effect of injection pressure of Naphtha on the (**a**) relative permeability, (**b**) saturation profile, and (**c**) fractional flow curve at GOR 2.5 MSCF/STB.

In this section, the effect of injection pressure on the performance of each rich gas was investigated. The effect of injection pressure on the performance of rich gas with LPG and NGL was the same as the effect of GOR. Both injection pressures did not have any influence on the output of the model (Figs. 13, 14). However, when gas was rich in Naphtha, the effect of injection pressure was clear. Higher injection pressure shifted the relative permeability of oil and gas to the right and left sides, respectively (Fig. 15). In addition, when the injection pressure was high, 2000 psi, gas moved faster in the medium, and the breakthrough occurred, while at two other injection pressures, 500 and 1500 psi, the breakthrough was not observed (Fig. 15). As shown in Fig. 15, the effect of injection pressure on the fractional flow curve was obvious. Higher injection pressure resulted in lower fractional flow of gas and consequently more oil will be produced.

## Conclusions

In this work, a new numerical simulation was developed to simulate the process of gas injection with rich gases, which can simulate the process in the three conditions: miscible, near miscible and immiscible. Effect of injection of rich gases, GOR of mixing process, and injection pressure on the output of the model were investigated. The main conclusions are summarized as follows:

1. Gas that is rich in Naphtha has lower IFT than two other rich gases because Naphtha is heavier than the two other ones.
2. As the main component of LPG and NGL is methane, the percentage of methane in these two rich gas was high; the IFT of LPG-oil and NGL-oil systems was close to IFT of methane-oil.
3. The GOR did not affect the performance of rich gas with LPG and NGL. Therefore, if LPG or NGL want to be used as an injection fluid for the reservoir that contains introduced crude oil, as GOR did not affected relative permeability of fluids, fractional flow, and saturation profile of gas, lower GOR was suggested.
4. In contrast to LPG and NGL, GOR affected the performance of naphtha. Decreasing the GOR shifted the relative permeability of oil to the right side and the relative permeability of gas to the left side. In addition, by decreasing GOR, gas moved fast in the domain. Therefore, for introduced oil, when naphtha was used as an injection fluid, in term of EOR, higher GOR of naphtha was resulted in better performance.
5. The same as GOR, injection pressure did not affect the performance of rich gas with NGL and LPG. While higher injection pressure resulted in faster movement of riched gas with Naphtha. Hence, lower injection pressure was suggested for injection fluid when naphtha was used as an injection fluid.

## Supplementary Information

Supplementary Information.

## Data Availability

All data generated or analysed during this study are included in this published article.

## Abbreviations

EOR: Enhanced oil recovery
GOR: Gas oil ratio
IFT: Interfacial tension
LPG: Liquefied petroleum gas
MMP: Minimum miscibility pressure
NGL: Natural gas liquid
WAG: Water alternative gas

## Symbols

$${CH}_{4}$$: Methane
$${CO}_{2}$$: Carbon dioxide
$${N}_{2}$$: Nitrogen
${y}_{g}$: Mole fraction of gas in the gas phase
${y}_{o}$: Mole fraction of oil in the gas phase
${x}_{o}$: Mole fraction of oil in the oil phase
${x}_{g}$: Mole fraction of gas in the oil phase
${M}_{o}$: Molecular weight of oil phase, $\frac{\mathrm{lbm}}{\mathrm{lbmol}}$
${M}_{og}$: Average molecular weight of oil phase, $\frac{\mathrm{lbm}}{\mathrm{lbmol}}$
${B}_{o}$: Oil formation volume factor, $\frac{\mathrm{bbl}}{\mathrm{STB}}$
${K}_{ro}^{imm}$: Immiscible oil relative permeability
${K}_{ro}$: Oil relative permeability at irreducible gas saturation
${K}_{RO}$: Oil relative permeability
${K}_{ro}^{mis}$: Miscible oil relative permeability
${S}_{or}^{imm}$: Residual oil saturation at the immiscible condition
${S}_{or}$: Residual oil saturation
${\rho }_{o}$: Density of oil phase, $\frac{\mathrm{lbm}}{{\mathrm{ft}}^{3}}$
${P}_{o}$: Parachor equation for oil phase
${\gamma }_{o}$: Specific gravity of oil
${\gamma }_{g}$: Specific gravity of gas
${\gamma }_{API}$: American Petroleum Institute
${\mu }_{oeff}$: Oil effective viscosity, $\mathrm{mPa s}$
${r}_{v}$: Vaporized oil in the gas phase, $\frac{\mathrm{scf}}{\mathrm{STB}}$
$${\mu }_{o}$$: Oil viscosity, $$\mathrm{mPa s}$$
${n}_{o}$: Oil exponent for Brooks–Corey functions
${x}_{g}$: Mole fraction of gas in the oil phase
${M}_{g}$: Molecular weight of gas phase, $\frac{\mathrm{lbm}}{\mathrm{lbmol}}$
${P}_{g}$: Parachor equation for gas phase
${\rho }_{g}$: Density of gas phase, $\frac{\mathrm{lbm}}{{\mathrm{ft}}^{3}}$
${M}_{go}$: Average molecular weight of gas phase, $\frac{\mathrm{lbm}}{\mathrm{lbmol}}$
$$Z$$: Compressibility factor
${R}_{s}$: Solution gas oil ratio, $\frac{\mathrm{scf}}{\mathrm{STB}}$
${K}_{rg}^{imm}$: Immiscible gas relative permeability
${K}_{RG}$: Gas relative permeability
${K}_{rg}^{mis}$: Miscible gas relative permeability
${K}_{rg}$: Gas relative permeability at residual oil saturation
${S}_{gi}^{imm}$: Irreducible gas phase saturation at the immiscible condition
${S}_{gi}$: Irreducible gas phase saturation
${\mu }_{geff}$: Gas effective viscosity, $\mathrm{mPa s}$
$${\mu }_{g}$$: Gas viscosity, $$\mathrm{mPa s}$$
$${\mu }_{m}$$: Mixing viscosity*, *$$\mathrm{mPa s}$$
$$\omega$$: Mixing factor
${n}_{g}$: Gas exponent for Brooks–Corey functions
${x}_{{S}_{g}}$: Distance moved by a specific ${S}_{g}$ contour, $\mathrm{m}$
$\frac{{df}_{g}}{{dS}_{g}}$: Derivative of the fractional flow of gas phase to gas saturation
${f}_{g}$: Fractional flow of gas
${F}_{k}$: Relative permeability interpolation parameter
${n}_{l}$: Read-in exponent
$${P}_{pc}$$: Pseudocritical pressure, $$\mathrm{psia}$$
$$P$$: Pressure, $$\mathrm{psia}$$
$${P}_{pr}$$: Pseudoreduced pressure
$${T}_{pc}$$: Pseudocritical temperature, $$\mathrm{R}$$
$$T$$: Temperature, $$\mathrm{R}$$
$${T}_{pr}$$: Pseudoreduced temperature
${n}_{m}$: Relative permeability index
${\sigma }_{go}$: IFT of gas and oil, $\frac{\mathrm{dynes}}{\mathrm{cm}}$
${\sigma }$: IFT at different pressure, $\frac{\mathrm{dynes}}{\mathrm{cm}}$
${\sigma }_{0}$: IFT at MMP, $\frac{\mathrm{dynes}}{\mathrm{cm}}$
$$\omega$$: Mixing factor
$Area$: Cross section area, ${\mathrm{m}}^{2}$
$$V$$: Viscosity ratio
${q}_{t}$: Total injection rate, $\frac{{\mathrm{m}}^{3}}{\mathrm{h}}$
$PVI$: Dimensionless pore volume
$$t$$: Injection time, $$\mathrm{h}$$
$L$: Length of domain, $\mathrm{m}$
$$\phi$$: Porosity, %
A-F: Brill and Beggs' constant for calculation of compressibility factors
