# Supplementary material for: Modeling of the movement of rich gas in a porous medium in immiscible, near miscible and miscible conditions
Source: Sci Rep. 2023 Apr 21;13:6573. doi: 10.1038/s41598-023-33833-5 (PMC10121694; doi:10.1038/s41598-023-33833-5)
Supplement: Supplementary file 1 — Supplementary Information. [file 41598_2023_33833_MOESM1_ESM.docx]

**Appendix**

Introduced parameters in Ramey's correlation are defined as follows:

| $M_{o}=6084/(\gamma_{API}-5.9)$ |  |
| --- | --- |
| $P_{o}=(2.376+0.0102\gamma_{API})M_{o}$ |  |
| $y_{o}={[1+\frac{7.521*{10}^{-6}M_{o}}{\gamma_{o}r_{v}}]}^{-1}$ |  |
| $x_{o}={[1+\frac{7.521*{10}^{-6}R_{s}M_{o}}{\gamma_{o}}]}^{-1}$ |  |
| $\rho_{o}=\frac{\gamma_{o}+2.179*{10}^{-4}\gamma_{g}R_{s}}{B_{o}}$ |  |
| $M_{g}=28.97*\gamma_{g}$ |  |
| $P_{g}=25.2+2.86M_{g}$ |  |
| $y_{g}=1-y_{o}$ |  |
| $x_{g}=1-x_{o}$ |  |
| $\rho_{g}=9.3184*{10}^{-2}\frac{pM_{go}}{62.4*ZT}$ |  |
| $M_{og}=x_{o}M_{o}+x_{g}M_{g}$ |  |
| $M_{go}=y_{o}M_{o}+y_{g}M_{g}$ |  |

Where, $M_{g}$ and $M_{o}$ are the molecular weight of gas and oil, respectively. The specific gravity of gas and oil are presented by $\gamma_{g}$ and $\gamma_{o}$ and by using $\gamma_{API}=\frac{141.5}{\gamma_{o}}-131.5$ specific gravity of oil and $\gamma_{API}$ can be related to each other. In the above equation, $r_{v}$ is used to show vaporized oil in the gas phase and as we used black oil model, $r_{v}=0$.
